# Measuring adult romantic attachment: psychometric properties of the brief Spanish version of the experiences in close relationships

**DOI:** 10.1186/s41155-020-00145-w

**Published:** 2020-06-05

**Authors:** Mónica Guzmán-González, Diana Rivera-Ottenberger, Audrey Brassard, Rosario Spencer, Marie-France Lafontaine

**Affiliations:** 1 School of Psychology, Universidad Católica del Norte, Avenida Angamos, 0610 Antofagasta, Chile; 2 School of Psychology, Pontificia Universidad Católica de Chile, Millennium Institute for Research in Depression and Personality (MIDAP), Avenida Vicuña Mackenna 4860, San Joaquín, Santiago, Chile; 3 Department of Psychology, University of Sherbrooke, 2500, boul. de l’Université, Sherbrooke, Québec, J1K 2R1 Canada; 4 Programa de Investigación Asociativa (PIA) en Ciencias Cognitivas, Centro de Investigación en Ciencias Cognitivas (CICC), Universidad de Talca, Avenida Lircay s/n, Talca, Chile; 5 School of Psychology, University of Ottawa, 136 Jean-Jacques Lussier, Ottawa, ON K1N6N5 Canada

**Keywords:** Adult attachment, Close relationships, Spanish version, Brief measure, Psychometric properties

## Abstract

The *Experiences in Close Relationships* (Brennan et al, Attachment theory and close relationships, 1998) questionnaire is one of the most widely used measures of adult romantic attachment. Despite the advantages of the ECR, the length of this measure may discourage its use in clinical and research contexts. Consequently, the goal of this study was to develop a brief Spanish version of the ECR questionnaire and to examine its psychometric properties when administered to six different Spanish-speaking samples from Chile. Confirmatory factor analyses replicated the two-dimensional structure of the ECR and its invariance across gender. Results also supported the reliability and concurrent validity of our brief Spanish version of the ECR (i.e., Spanish ECR-12), by its association with measures of emotion regulation, dyadic empathy, psychological distress and well-being, and relationship satisfaction. The Spanish ECR-12 can be used by researchers and clinical professionals as an abridged measure of adult attachment.

## Introduction

Attachment theory articulates the universal human need to create close emotional bonds that can provide comfort and protection during times of stress and pain (Bowlby, 1982). According to this theory, the quality of interactions with one’s attachment figures in childhood impacts early internal representations of the self and others, which, in turn, influence affect, cognition, and behavior. These internal models can affect the quality of close relationships throughout the lifespan (Mikulincer & Shaver, 2016).

Adults with greater attachment security tend to view themselves as worthy of love and feel comfortable depending on others, while adults with greater attachment insecurity tend to perceive themselves and/or others negatively (Bartholomew & Horowitz, 1991). Insecure adult attachment can be conceptualized according to two dimensions known as attachment anxiety and attachment avoidance (Brennan, Clark, & Shaver, 1998). Attachment anxiety is characterized by the degree to which individual feel that they do not deserve affection, care, and protection, which could lead to chronic doubt about their standing in a relationship due to their persistent concern about being abandoned or rejected and their excessive distress when their partner is unresponsive (Mikulincer & Shaver, 2016). Attachment avoidance is characterized by the degree to which an individual considers others as unavailable in times of need, which could lead to exacerbated self-reliance, discomfort with closeness and self-disclosure, and difficulty in trusting others (Mikulincer, Shaver, & Slav, 2006). Based on this conceptualization, attachment insecurity is characterized by either higher levels of attachment anxiety and/or attachment avoidance.

It is well documented that insecure romantic attachment is associated with poor outcomes in intrapersonal functioning and couple functioning (see Mikulincer & Shaver, 2016, for a review). In terms of intrapersonal functioning, attachment insecurity has been associated with mental health problems, including anxiety and depression (Zakalik & Wei, 2006), lower levels of life satisfaction (Guzmán-González et al., 2016a), and emotion regulation difficulties (Brenning & Braet, 2013; Guzmán-González, Lafontaine, & Levesque, 2016; Pascuzzo, Cyr, & Moss, 2013). In terms of couple functioning, attachment insecurity has been associated with lower levels of relationship satisfaction (Feeney, 2016; Li & Chan, 2012), both in heterosexual (e.g., Birnbaum, 2007; Butzer & Campbell, 2008; Guzmán & Contreras, 2012; Heresi-Milad, Rivera-Ottenberger, & Huepe-Artigas, 2014) and same-sex couples (Horne & Biss, 2009). Attachment insecurity has also been related to lower empathy for others (Wei, Liao, Ku, & Shaffer, 2011), including a romantic partner (Lafontaine, Guzmán-González, Péloquin, & Levesque, 2016; Péloquin, Lafontaine, & Brassard, 2011).

## Measures of adult romantic attachment

Based on the seminal work of Hazan and Shaver (1987), who applied the principles of the attachment theory to romantic relationships, a vast number of self-report measures were developed, such as the *Adult Attachment Questionnaire* (Simpson, 1990), the *Adult Attachment Scale* (Collins & Read, 1990), and the *Attachment Style Questionnaire* (Feeney, Noller, & Hanrahan, 1994). Brennan et al. (1998) attempted a factor reduction of the various items available on all attachment measures in use at the time and identified two major factors common to most of these instruments, namely, attachment avoidance and attachment anxiety. Based on these results, they used the best 36 items to create the *Experiences in Close Relationships* (ECR; Brennan et al., 1998). To our knowledge, none of the questionnaires that were used to create the ECR has been adapted in Spanish, except for the *Adult Attachment Scale* (Fernández & Dufey, 2015).

Since the work led by Brennan et al. (1998), the two-dimensional conceptualization of adult attachment has progressively been the most recommended and prevalent way to capture variations in attachment over categorical classifications (Mikulincer & Shaver, 2016). Categorical measures are criticized due to a variety of reasons, including the fact that classifications are not stable over time, they are often treated as mutually exclusive, and they rely on the assumption that differences among people within a category are irrelevant (Gillath, Karantzas, & Fraley, 2016; Mikulincer & Shaver, 2016). The use of continuous measures is preferred because they address those limitations, capturing variability in each attachment dimension, leading to greater reliability.

Defended by Mikulincer and Shaver (2016), the ECR is the gold standard for measuring romantic attachment (i.e., attachment-related anxiety and avoidance) because of its good psychometric properties (i.e., high reliability and validity) and its validation for use in different cultures and languages. The Spanish version of the ECR has been validated in numerous Spanish-speaking populations (e.g., Alonso-Arbiol, Balluerka, & Shaver, 2007), including Chilean samples (Spencer, Guzmán, Fresno, & Ramos, 2013).

Despite the advantages of the ECR, the length of this measure (i.e., 36 items; 18 per dimension) may discourage its use in clinical and research contexts. An abridged version of the ECR would make it feasible to include the ECR in studies requiring a large battery of tests (Spencer, Alonso-Arbiol, & Fresno, 2013). To this day, there are two brief English versions of the ECR; both comprise 12 items. The *ECR Scale Short Form* (ECR-S; Wei, Russell, Mallinckrodt, & Vogel, 2007) is the result of six studies. The results of Wei et al.’s (2007) studies indicate that the ECR-S has good psychometrics properties (i.e., internal consistency, test-retest reliability, factor structure, and validity), comparable or equivalent to the original ECR. However, while Lafontaine et al. (2016a) acknowledged the contribution of ECR-S, they raised three limitations that justified the creation of a new abridged version of the original ECR. First, the ECR-S has reduced generalizability due to the characteristic of their sample (i.e., college students). Second, the factor structure is acceptable only after controlling for two additional latent variables accounting for response sets, which was not applicable to the original ECR. Third, they argued that the classical test theory, which was used to select the final scale items, has many statistical inconveniences. To overcome these difficulties, Lafontaine et al. (2016a) conducted five studies to create the ECR-12. Using item response theory, their studies demonstrated the good psychometric properties of the ECR-12 in different samples (i.e., students, community, clinical, and same-sex couples), preserving the good psychometrics properties of the original ECR and surpassing the ECR-S. Although two abridged English versions of the ECR exist, to our knowledge, there is only one brief Spanish version of the ECR (Chilean B-ECR; Spencer, Alonso-Arbiol et al., 2013a). Even though the Chilean B-ECR has good psychometric properties, it presents three limitations mentioned by the authors. First, the sample used is not generalizable to the entire population of Spanish-speaking individuals. Second, concurrent validity has not been evaluated. Third, this version is still lengthy (i.e., 25 items). Despite criticisms both on the extent and specificity of the evaluation of adult attachment (Fraley, Heffernan, Vicary, & Brumbaugh, 2011), the present study aims to provide a psychometrically sound abridged Spanish version of the ECR for the study of relationships in Spanish-speaking Chilean populations that has psychometric properties comparable to the original ECR (Brennan et al., 1998).

## The present research

Given the importance of readily accessible brief scales of important constructs, such as romantic attachment, in different research contexts that are also suitable for a clinical setting, our general goal was to obtain an abridged Spanish version of the ECR that would be psychometrically sound for use with diverse Chilean populations. More specifically, our first goal was to test the factorial structure of the three existing brief versions of the ECR, given the lack of information available regarding the appropriateness of these versions for our data. The ECR-S (Wei et al., 2007), ECR-12 (Lafontaine et al., 2016a), and Chilean B-ECR (Spencer, Alonso-Arbiol et al., 2013a) proposed a two-factor model (i.e., attachment anxiety and attachment avoidance). We thus aim to test these three bidimensional structures on five independent samples of adults who had been involved in a couple relationship at least once in their lifetime: two student samples (samples 2 and 3), a community sample (sample 4), a community sample of gay and lesbian adults (sample 5), and a community sample of heterosexual couples (sample 6). As these three brief ECR versions may not be an appropriate representation of Chilean adults’ romantic attachment, our second goal was to develop a new brief version that would be comparable to the original ECR (Brennan et al., 1998), based on another large sample of Chilean adults from the community (sample 1). An exploratory factor analysis (EFA) approach was favored to select the best items assessing attachment anxiety and attachment avoidance on the existing 36-item Chilean ECR version (Spencer, Guzmán, et al., 2013b).

Our third goal was to examine the psychometric properties of the new brief version of the ECR scale, using all six samples of adults. Based on the results from Del Giudice’s (2011) meta-analysis, which suggested that men display higher avoidance and lower anxiety than women, we also examined possible gender differences in men and women’s responses (i.e., gender metric invariance) in our new brief Spanish ECR. The questions we anticipated to answer in the present study were as follows: RQ1: Will the new brief Spanish ECR be an appropriate representation of a population of Chilean adults involved in a couple relationship?; RQ2: Will the internal consistency of the new brief Spanish ECR be comparable with the 36-item Chilean ECR (Spencer, Guzmán, et al., 2013b?; RQ3: Will the romantic attachment dimensions of the brief Spanish ECR be correlated with the same dimensions of the 36-item Chilean ECR?; RQ4 to RQ7: Will there be an association between (a) emotion regulation difficulties, dyadic empathy, psychological distress, as well as individual and relationship well-being, and (b) attachment anxiety and attachment avoidance?

## Method

### Participants

We included a total of 3703 Spanish-speaking Chilean adults, coming from six independent samples. In addition to students (samples 2 and 3), adults from the community (samples 1 and 4), gay and lesbian adults (sample 5), and heterosexual couples from the community (sample 6) were included to increase the representability of our results and to validate the scale among several populations. Demographic characteristics of the six samples are presented in Table 1.

### Procedure

The ethical components of the studies included here (or in the current manuscript) were approved by the Ethics Committee of the involved institutions. Participants from the six samples were recruited using a variety of methods, for example, snowball sampling, contacted by trained research assistants, and email listserv announcements. Data were collected in different cities in Chile (e.g., Antofagasta, Valparaíso, Santiago, Talca, Concepción). For all six samples, informed consent was obtained, and participants completed a paper-and-pencil version of the 36-item ECR Chilean version (Spencer, Guzmán, et al., 2013b) and additional validity scales assessing various attachment-related theoretical constructs. As six independent samples were used, some questionnaires were completed in some samples and not others; this is because the studies behind those samples were targeting different objectives.

### Measures

All scales were chosen based on their psychometric properties and past empirical associations with attachment insecurities. Cronbach’s alpha coefficients for these measures and their respective subscales are displayed in Table 1. Participants from all six samples completed a short demographic questionnaire, including items about their age, gender, and relationship status.

**Table 1:** Demographic features of the six samples

				Age		
Sample	*N*	Women (%)	Men (%)	*M*	*SD*	Participants in a current relationship (%)
1 (Community A)	943	48.9	51.1	38.80	13.75	73.4
2 (Students A)	919	53.5	46.5	21.37	2.23	60.7
3 (Students B)	578	55.4	44.6	21.86	2.76	74.6
4 (Community B)	436	47.9	52.1	39.39	13.43	55.7
5 (Gay/lesbian)	467	42.6	57.4	27.87	7.89	55.9
6 (Female partner)	180	100.0	0.0	44.59	9.46	100.0
6 (Male partner)	180	0.0	100.0	47.31	10.24	100.0

### Emotion regulation difficulties

The *Difficulties in Emotion Regulation Scale* (DERS; Gratz & Roemer, 2004), which was validated for use in a Chilean population by Guzmán-González, Trabucco, Urzúa, Garrido, and Leiva (2014), assesses emotion regulation difficulties. The DERS Chilean version comprises 25 items, which are evaluated on a 5-point scale, with responses ranging from 1 (*almost never*) to 5 (*almost always*). Examples of items are “I have difficulty making sense out of my feelings” and “I experience my emotions as overwhelming and out of control*.*” Higher scores indicate greater difficulties in emotional regulation. This instrument includes five subscales, with reported Cronbach's alpha coefficients ranging from .66 to .89. The five dimensions were averaged in a global score of emotional regulation. The DERS was used in samples 1, 2, and 5.

### Dyadic empathy

The *Interpersonal Reactivity Index for Couples* (IRIC; Péloquin & Lafontaine, 2010), which was validated for use in a Chilean population by Guzmán-González, Péloquin, Lafontaine, Trabucco, and Urzúa (2014), assesses two aspects of dyadic empathy: perspective taking (e.g., “I sometimes try to understand my partner better by imagining how things look from his/her perspective”) and empathic concern (e.g., “I often have tender, concerned feelings for my partner when he/she is less fortunate than me”). The IRIC Chilean version comprises 13 items, which are evaluated on a 5-point scale, with responses ranging from 0 (*does not describe me well*) to 4 (*describes me very well*). All items are summed to obtain the global score. Higher scores indicate greater perspective taking and empathic concern. Cronbach’s alpha coefficients have been reported to be .58 and .82 for empathic concern and perspective taking subscales, respectively (Guzmán-González et al., 2014). This instrument was used in samples 1 and 2.

### Psychological distress

Psychological distress was measured with three instruments:

The *Outcome Questionnaire* (OQ-45; Lambert et al., 1996), which was validated for use in a Chilean population (von Bergen & de la Parra, 2002), assesses outcome in psychotherapy in three areas: symptom distress, interpersonal relations, and social role. The OQ-45 comprises 45 items, which are evaluated on a 5-point scale, with responses ranging from 0 (*never*) to 4 (*almost always*). Examples of items are “I feel no interest in things” and “I feel lonely.” Higher scores indicate greater symptomatic discomfort. The validated Chilean version of the OQ-45 has excellent internal consistency, with Cronbach’s alpha coefficient of .91. This instrument was used in sample 1 as an indicator of psychopathology.

The *Abbreviated Scales of Depression, Anxiety, and Stress* (DASS-21; Lovibond & Lovibond, 1995), which was validated for use in a Chilean population by Vinet, Rehbein, Román, and Saiz (2008), assesses three aspects of mental health: depression, anxiety, and stress. The DASS-21 comprises 21 items, which are evaluated on a 4-point scale, with responses ranging from 0 (*it does not describe anything that occurred to me or that I felt during the week*) to 3 (*yes, it occurred to me a lot, or almost always*). Examples of items are “I found it difficult to relax” and “I found it difficult to work up the initiative to do things.” Higher scores indicate more symptomatology. Cronbach’s alpha coefficients obtained by the authors of the validated Chilean version were .85, .83, and .73 for the depression, anxiety, and stress subscale, respectively. This instrument was used in sample 4.

The *Center for Epidemiologic Studies Depression Scale* (CES-D; Radloff, 1977) Spanish version (Gempp, Avendaño, & Muñoz, 2004) assesses depressive symptoms in the general population. The CES-D comprises 20 items, which are evaluated on a 4-point scale, with responses ranging from 0 (*rarely/never (1 day or less)*) to 3 (*most/all days (5–7 days)*). Examples of items are “I felt that everything I did was an effort” and “My sleep was restless.” Higher scores indicate more depressive symptoms. The CES-D Spanish version has good internal consistency, with a Cronbach's alpha coefficient of .87. This instrument was used in sample 6.

### Individual and relationship well-being

The *Satisfaction with Life Scale* (SLS; Diener, Emmons, Larsen, & Griffin, 1985), validated in Chile by Vera-Villarroel, Urzúa, Pavez, Celis-Atenas, and Silva (2012) assesses life satisfaction. The SLS comprises five items, which are evaluated on a 7-point Likert scale, with responses ranging from 1 (*completely disagree*) to 7 (*completely agree*). Example of items are “In most ways my life is close to my ideal” and “I am satisfied with my life.” Higher scores indicate greater satisfaction with life. This instrument has good internal consistency, with Cronbach’s alpha coefficient of .87 (Vera-Villarroel et al., 2012). This instrument was used in samples 4 and 5.

The *Relationship Assessment Scale***(**RAS, Hendrick, 1988) Spanish version (Rivera, Cruz, & Muñoz, 2011) assesses relationship satisfaction. The RAS comprises seven items, which are evaluated on a 5-point scale, with responses ranging from 1 (*low satisfaction*) to 5 (*high satisfaction*). Examples of items are “How well does your partner meet your needs?” and “How good is your relationship compared to most?” In Chilean samples, Cronbach’s alpha coefficient is .71 (Rivera et al., 2011). This instrument was used in sample 6.

### Data analysis strategy

First, confirmatory factor analyses (CFA) were conducted on samples 2, 3, 4, 5, and 6 to test the factorial structure of existing brief versions of the ECR. We tested the models separately for men and women from sample 6 to account for the non-independence of the dyadic data. Based on a meta-analysis suggesting a stable correlation between the ECR attachment anxiety and avoidance subscales (Cameron, Finnegan, & Morry, 2012), we estimated the covariance between the two factors. We tested each CFA using several fit indices, following the recommendation of Kline (2015). The comparative fit index (CFI) and Tucker-Lewis index (TLI) values typically range from 0 to 1, and values equal to or greater than .90 are representative of an adequate fitting model (with values close to .95 or higher indicate a model that fits the data well). The root mean square error of approximation (RMSEA) calculates the average error of fit between the submitted model and the data, and a value equal to or lower than .08 corresponds to an adequate fit (Browne & Cudeck, 1993). The close fit hypothesis is supported if a 90% confidence interval includes the .05 value and the poor fit hypothesis is supported if the upper bound of the confidence interval does not exceed a .10 value (Kline, 2016). We used the *χ*^2^/*df* ratio because the chi-square test is perceived to be less valid with a larger sample size. A ratio value between 1 and 5 suggests a satisfactory fit between the proposed theoretical model and the data (Bollen, 1989).

Second, two-factor exploratory factor analyses (EFA) with maximum likelihood extraction and oblimin rotation were conducted on an independent sample (sample 1) to select the 12 best items for a psychometrically sound short version of the Spanish ECR. A Kaiser-Meyer-Olkin (KMO) of .80 and higher indicated good sample adequacy and significant Bartlett’s test of sphericity supported the presence of correlations. Item selection was based on four criteria: (a) factor loadings, (b) item-total correlation, (c) low cross-loading (*r* < .30), and (d) content validity (i.e., covering the theoretical components of each attachment subscale).

Third, to assess the psychometric properties of the new brief Spanish ECR, CFA were conducted on samples 2 to 6, followed by gender metric invariance analyses (i.e., comparing the equivalence of factor loadings between men and women). Pearson correlation analyses were then conducted between the short and original version to assess the representativeness of our new subscales as well as Cronbach’s alpha coefficients, with 95% confidence intervals. Finally, we conducted multiple regression analyses on a series of validity criteria to examine the concurrent validity of the Spanish ECR-12. As recommended by Cameron et al. (2012), each analysis included both attachment anxiety and attachment avoidance as predictors to account for their shared variance. The same analyses were carried out with the respective original ECR subscale and *t* tests assessed the presence of significant differences between the standardized regression coefficients (original ECR vs Spanish ECR-12 subscales).

## Results

### Step 1: Testing the factorial structure of the existing brief versions of the ECR

Using samples 2 to 6, we conducted CFA to test the three existing brief versions of the ECR: the French/English ECR-12 (Lafontaine et al., 2016a), the English ECR-S (Wei et al., 2007), and Chilean B-ECR (Spencer et al., 2013a). The results (see Table 2) suggested poor fit for all three abridged versions, supporting our choice to select better items for a psychometrically sound new Brief Spanish ECR.

**Table 2:** Cronbach’s alpha for the validity criteria and standardized regression coefficients from analyses in which ECR subscales predicted these criteria across samples

Validity criteria	Cronbach *α*	ECR-12 anxiety	ECR anxiety	ECR-12 avoidance	ECR avoidance
Sample 1					
DERS – Emotion Regulation Difficulties	.92	.30***	.40***	.34***	.30***
OQ-45 Total	.91	.24***	.31***	.32***	.32***
IRIC Perspective-Taking	.83	.03	.05	−.25***	−.37***
IRIC Empathic Concern	.61	.29***	.28***	−.31***	−.39***
Sample 2					
DERS – Emotion Regulation Difficulties	.91	.33***	.42***	.27***	.28***
IRIC Perspective-Taking	.81	−.10**	−.13***	−.27***	−.30***
IRIC Empathic Concern	.55	.30***	.25***	−.36***	−.41***
Sample 4					
SLS Life Satisfaction	.81	−.20***	−.26***	−.25***	−.31***
DASS-21 Depression	.87	.28***	.33***	.21***	.21***
DASS-21 Anxiety	.84	.20***	.23***	.20***	.19***
DASS-21 Stress	.89	.30***	.33***	.16**	.16**
Sample 5					
DERS – Emotion Regulation Difficulties	.92	.30***	.39***	.31***	.29***
SLS Life Satisfaction	.80	−.23***	−.28***	−.13**	−.16**
Relationship Satisfaction	.87	−.09*	−.15***	−.48***	−.57***
Sample 6					
Relationship Satisfaction W	.91	−.05	−.07	−.54***	.66***
CES-D Depression W	.89	.27***	.30***	.14	.14
Relationship Satisfaction M	.89	−.07	−.07	−.29***	−.45***
CES-D Depression M	.86	.29***	.31***	.17*	.18*

*Note*: Coefficients that significantly differ from each other (*p* < .01) are in boldface. *W* women, *M* Men, *DERS* Difficulties in Emotion Regulation Scale, *OQ* Outcome Questionnaire, *IRIC* Interpersonal Reactivity Index for Couples, *DASS* Depression Anxiety Stress Scale, *LSS* Life Satisfaction Scale, *CES-D* Center for Epidemiologic Studies Depression Scale

*p < .05. **p < .01. ***p < .001

### Step 2: Item selection process for the new brief Spanish ECR

Using a sample of 943 community-based Chilean adults (see sample 1 in Table 3), a two-factor EFA was conducted on the 36-item Chilean ECR version (Spencer, Guzmán, et al., 2013b). The KMO of .92 indicated good sample adequacy and Bartlett’s test of sphericity, *χ*^2^ (630, *N* = 943) = 12357.99, *p* < .001, supported the presence of correlations. The two-factor solution explained 36.43% of the shared variance from the 36 items. We then proceeded to select the best six items from the attachment anxiety and attachment avoidance subscale based on four criteria: (1) high factor loadings (see Table 4), (2) high item-total correlation (see Table 4), (3) low cross-loading (*r* < .30), and (4) content validity.

**Table 3:** Fit indexes for the confirmatory factor analyses of the Spanish ECR-12, French/English ECR-12, English ECR-S, and Chilean B-ECR

Sample	*χ*^2^	*χ*^2^/*df*	CFI	TLI	RMSEA [90% CI]
**2 (Students A)**	***χ***^**2**^**(52,*N*= 919) = 156.07,*p*< .001**	**3.00**	**.95**	**.94**	**.05 [.04; .06]**
ECR-12	*χ*^2^(52, *N* = 919) = 340.09, *p* < .001	6.54	.85	.81	.08 [.07; .09]
ECR-S	*χ*^2^(53, *N* = 919) = 610.78, *p* < .001	11.52	.70	.63	.11 [.10; .12]
B-ECR	*χ*^2^(274, *N* = 919) = 1747.89, *p* < .001	6.38	.76	.74	.08 [.07; .08]
**3 (Students B)**	***χ***^**2**^**(52,*N*= 578) = 160.19,*p*< .001**	**3.08**	**.95**	**.94**	**.06 [.05; .07]**
ECR-12	*χ*^2^(52, *N* = 578) = 281.70, *p* < .001	5.42	.90	.90	.09 [.08; .10]
ECR-S	*χ*^2^(53, *N* = 578) = 610.78, *p* < .001	11.52	.70	.63	.11 [.10; .12]
B-ECR	*χ*^2^(274, *N* = 578) = 1737.39, *p* < .001	6.34	.76	.74	.10 [.09; .10]
**4 (Community)**	***χ***^**2**^**(52,*N*= 436) = 146.20,*p*< .001**	**2.81**	**.95**	**.93**	**.06 [.05; .08]**
ECR-12	*χ*^2^(52, *N* = 436) = 163.12, *p* < .001	3.14	.92	.89	.07 [.06; .08]
ECR-S	*χ*^2^(53, *N* = 436) = 337.83, *p* < .001	6.37	.74	.67	.11 [.10; .12]
B-ECR	*χ*^2^(274, *N* = 436) = 1173.82, *p* < .001	4.28	.79	.77	.09 [.08; .09]
**5 (Gay/lesbian)**	***χ***^**2**^**(52,*N*= 467) = 104.47,*p*< .001**	**2.01**	**.97**	**.96**	**.05 [.03; .06]**
ECR-12	*χ*^2^(52, *N* = 467) = 177.76, *p* < .001	3.42	.91	.86	.07 [.06; .08]
ECR-S	*χ*^2^(53, *N* = 467) = 292.63, *p* < .001	5.52	.80	.70	.10 [.09; .11]
B-ECR	*χ*^2^(274, *N* = 467) = 1046.87, *p* < .001	3.82	.81	.77	.08 [.07; .08]
**6 (Female partner)**	***χ***^**2**^**(52,*N*= 180) = 102.32,*p*< .001**	**1.97**	**.92**	**.90**	**.08 [.06; .10]**
ECR-12	*χ*^2^(52, *N* = 180) = 127.30, *p* < .001	2.45	.85	.78	.09 [.07; .11]
ECR-S	*χ*^2^(53, *N* = 180) = 176.87, *p* < .001	3.34	.78	.68	.11 [.10; .13]
B-ECR	*χ*^2^(274, *N* = 180) = 679.57, *p* < .001	2.48	.77	.73	.09 [.08; .10]
**6 (Male partner)**	***χ***^**2**^**(52,*N*= 180) = 87.54,*p*< .001**	**1.68**	**.94**	**.93**	**.06 [.04; .08]**
ECR-12	*χ*^*2*^(52, *N* = 180) = 171.41, *p* < .001	3.29	.76	.64	.11 [.10; .13]
ECR-S	*χ*^2^(51, *N* = 180) = 194.83, *p* < .001	3.68	.70	.56	.12 [.10; .14]
B-ECR	*χ*^2^(51, *N* = 180) = 833.30, *p* < .001	3.04	.45	.58	.11 [.10; .12]

*Note*: Samples and statistics for the Spanish ECR-12 are presented in boldface

**Table 4:** Saturation coefficients and item-total correlations for the 36 items in sample 1

Item	Anxiety	Avoidance	Item-total *r*
1. I prefer not to show a partner how I feel deep down. | Prefiero no mostrar a mi pareja mis sentimientos personales.	.127	.505	.475
3. I am very comfortable being close to romantic partners. | Me siento muy cómodo/a teniendo un alto grado de intimidad con mi pareja.	−.131	.551	.488
5. Just when my partner starts to get close to me I find myself pulling away. | Cuando mi pareja comienza a establecer mayor intimidad, me doy cuenta que tiendo a cerrarme.	.203	**.627**	**.595**
7. I get uncomfortable when a romantic partner wants to be very close. | Me siento muy irritado/a cuando mi pareja quiere demasiada intimidad emocional.	.232	.530	.499
**9. I don’t feel comfortable opening up to romantic partners. | Me siento incómodo/a abriéndome a mi pareja.**	.198	**.608**	**.568**
*11. I want to get close to my partner, but I keep pulling back. | Quiero acercarme afectivamente a mi pareja, pero a la vez pongo distancia entre nosotros.*	.320	.561	.536
*13. I am nervous when partners want to get too close to me. | Me pongo nervioso/a cuando mi pareja logra demasiada intimidad emocional conmigo.*	.277	**.598**	**.558**
**15. I feel comfortable sharing my private thoughts and feelings with my partner. ® | Me siento cómodo/a compartiendo mis sentimientos y pensamientos más íntimos con mi pareja.**	.155	−.501	.451
*17. I try to avoid getting too close to my partner. | Intento evitar establecer demasiada intimidad con mi pareja.*	.192	**.605**	**.553**
19. I find it relatively easy to get close to my partner. ® | Encuentro relativamente fácil establecer intimidad emocional con mi pareja.	.098	−.525	.479
21. I find it difficult to allow myself to depend on romantic partners. | Me es difícil permitirme depender de mi pareja.	.102	.131	.148
23. I prefer not to be too close to romantic partners. | Prefiero no tener demasiada intimidad emocional con mi pareja.	.152	**.659**	**.611**
**25. I tell my partner just about everything. ® | Se lo cuento todo a mi pareja.**	.218	−.494	.457
***227. I usually discuss my problems and concerns with my partner. ® | Frecuentemente converso sobre mis problemas y preocupaciones con mi pareja.***	.114	−**.594**	**.554**
**29. I feel comfortable depending on romantic partners. ® | Me siento cómodo/a dependiendo de mi pareja.**	.327	−.140	.114
**31. I don’t mind asking romantic partners for comfort, advice, or help. ® | No me complica pedirle a mi pareja que me ayude, me consuele o me aconseje.**	.168	−.524	.471
*33. It helps to turn to my romantic partner in times of need. ® | Me ayuda mucho recurrir a mi pareja en épocas de crisis.*	.236	−.540	.491
*35. I turn to my partner for many things, including comfort and reassurance. ® | Recurro a mi pareja para muchas cosas, por ejemplo cuando necesito consuelo y tranquilidad.*	.293	−.589	.531
**2. I worry about being abandoned. | Me preocupa que me abandonen.**	.561	−.049	.553
4. I worry a lot about my relationships. | Me preocupo en exceso por mis relaciones.	.543	−.126	.501
***6. I worry that romantic partners won’t care about me as much as I care about them. | Me preocupa que mi pareja no se interese por mí tanto como yo me intereso por ella.***	**.599**	.048	**.578**
**8. I worry a fair amount about losing my partner. | Me preocupa bastante la posibilidad de perder a mi pareja.**	**.595**	−.096	**.582**
10. I often wish that my partner’s feelings for me were as strong as my feelings for him / her. | Frecuentemente deseo que los sentimientos de mi pareja hacia mí sean tan fuertes como son mis sentimientos hacia él/ella.	.548	−.095	.498
12. I often want to merge completely with romantic partners, and this sometimes cares them away. | Frecuentemente quisiera fusionarme completamente con mi pareja y esto a veces le asusta.	.523	.261	.521
**14. I worry about being alone. | Me preocupa estar solo/a.**	.560	−.004	.562
*16. My desire to be very close sometimes scares people away. | A veces mi deseo de excesiva intimidad asusta a la gente.*	.391	.177	.393
***18. I need a lot of reassurance that I am loved by my partner. | Necesito que me pareja me reafirme constantemente que me ama.***	**.630**	−.123	**.563**
20. Sometimes I feel that I force my partners to show more feeling, more commitment. | A veces siento que presiono a mi pareja para que muestre más sentimientos, más compromiso hacia nuestra relación.	**.598**	.142	**.568**
*22. I do not often worry about being abandoned. ® | Pocas veces me preocupa la idea de ser abandonado/a.*	−.158	.049	.177
**24. If I can’t get my partner to show interest in me, I get upset or angry. | Si no logro que mi pareja muestre interés por mí, me molesto o me enojo.**	**.608**	.127	**.576**
*26. I find that my partner(s) don’t want to get as close as I would like. | Creo que mi pareja no quiere tener tanta intimidad emocional conmigo como a mí me gustaría.*	.473	.354	.495
28. When I’m not involved in a relationship, I feel somewhat anxious and insecure. | Cuando no tengo una relación de pareja, me siento un poco ansioso/a e inseguro/a.	.501	.137	.495
30. I get frustrated when my partner is not around as much as I would like. | Me siento frustrado/a cuando mi pareja no me hace tanto caso como a mí me gustaría.	**.679**	.075	**.644**
*32. I get frustrated if romantic partners are not available when I need them. | Me siento frustrado/a si mi pareja no está disponible cuando la necesito.*	.632	−.017	.583
34. When romantic partners disapprove of me, I feel really bad about myself. | Cuando mi pareja me critica, me siento muy mal.	.504	.040	.459
36. I resent it when my partner spends time away from me. | Me molesta que mi pareja pase tiempo lejos de mí.	.596	−.097	.542

*Note*: Items retained in the Spanish ECR-12 are highlighted. Items retained in the ECR-12 (Lafontaine et al., 2016) are in boldface. Items retained in the ECR-S (Wei et al., 2007) are in italics

Based on these criteria, the six items selected for attachment anxiety were representative of its three theoretical components: fear of interpersonal rejection (items 6, 8), disproportionate need for approval from others (items 18, 20), and distress when one’s partner is unavailable (items 24, 30). Although item 32 presented high factor loading and item-total correlation, it was discarded because of its high correlation and similar wording with item 30. The six items selected for attachment avoidance were representative of two out of three theoretical components: fear of interpersonal intimacy (items 5, 13, 17, 23) and reluctance to self-disclose (items 9, 27). Excessive need for self-reliance, however, was not represented in the selected items.

A second two-factor EFA was conducted on the selected 12 items. The KMO of .87 indicated good sample adequacy and Bartlett’s test of sphericity, *χ*^2^ (66, *N* = 943) = 3424.29, *p* < .001, supported the presence of correlations. The two-factor solution explained 42.98% of the shared variance from the 12 items. As expected, attachment anxiety and attachment avoidance items showed high factor loadings (> .40) on their respective factors and low factor loadings on the other factor (< .20).

### Step 3: Psychometric properties of the new ECR

#### Factorial structure

Confirmatory factor analyses (CFA) were conducted on samples 2 to 6 to confirm the factor structure of the Spanish ECR-12 across diverse Chilean populations. Maximum likelihood estimation was employed to estimate the two-factor model, with items 6, 8, 18, 20, 24, and 30 predicted by the Spanish ECR-12 anxiety latent factor and items 5, 9, 13, 17, 23, and 27 predicted by the Spanish ECR-12 avoidance latent factor. A covariance was also added between the error terms of two anxiety items (i.e., 6 and 8) to account for similar wording. As shown in Table 2, the indexes of fit were adequate for the Spanish ECR-12. The correlations between the two latent factors ranged from *r* = .24 (*p* < .001) in sample 5 to *r* = .42 (*p* < .001) in male partners from sample 6, suggesting a weak to moderate association. In sample 3, however, the correlation was null, *r* = .04 (*p* = .447).

#### Gender metric invariance

Comparison of the equivalence of factor loadings between men and women were conducted, using the metric invariance test on Mplus. Gender invariance testing revealed no significant difference between men and women in the factor loadings in sample 2, Δ*χ*^2^(10, *N* = 919) = 10.59, *p* = .390; sample 3, Δ*χ*^2^(10, *N* = 578) = 8.99, *p* = .532; sample 4, Δ*χ*^2^(10, *N* = 436) = 7.65, *p* = .663; and sample 6, Δ*χ*^2^(10, *N* = 180) = 14.93, *p* = .135. Gender invariance, however, was not obtained between gay and lesbian from sample 5, Δ*χ*^2^(10, *N* = 467) = 27.87, *p* = .002. Further analysis revealed that partial metric invariance was supported; specifically, items 9 and 27 from the avoidance subscale showed higher factor loadings in gay men compared to women.

#### Correlations between the ECR subscales

To ensure the representativeness of the Spanish ECR-12, we conducted Pearson correlations with 95% confidence intervals between the brief and the original subscales of the 36-item Chilean ECR version (Spencer, Guzmán, et al., 2013b). The results, presented in Table 5, supported the presence of very high correlations between the brief attachment anxiety subscale and the original attachment anxiety subscale, with *r* coefficients ranging from .89 to .93. We also found very high correlations between the brief attachment avoidance subscale and the original attachment avoidance subscale, with *r* coefficients ranging from .85 to .92.

**Table 5:** Means, standard deviations, and Cronbach’s alpha and correlation coefficients with 95% confidence intervals for ECR anxiety and avoidance subscales

	Anxiety					Avoidance				
	Spanish ECR-12			ECR	Subscales	Spanish ECR-12			ECR	Subscales
Sample	*M*	*SD*	*α*	*α*	*r*	*M*	*SD*	*α*	*α*	*r*
1	3.73	1.36	.80 [.78; .82]	.89 [.88; .90]	.91 [.90; .92]	2.49	1.20	.82 [.80; .83]	.86 [.85; .87]	.88 [.87; .90]
2	3.99	1.20	.72 [.70; .75]	.84 [.83; .86]	.89 [.88; .91]	2.44	1.14	.78 [.75; .80]	.85 [.83; .86]	.88 [.86; .90]
3	4.02	1.39	.83 [.80; .85]	.90 [.89; .91]	.92 [.91; .93]	2.34	1.15	.84 [.82; .86]	.89 [.88; .91]	.92 [.90; .93]
4	3.67	1.35	.82 [.79; .84]	.89 [.87; .90]	.93 [.92; .94]	2.54	1.23	.83 [.80; .85]	.84 [.82; .87]	.88 [.84; .91]
5	3.74	1.46	.83 [.80; .85]	.90 [.89; .91]	.93 [.92; .94]	2.20	1.14	.79 [.75; .81]	.85 [.83; .87]	.89 [.86; .91]
6 W	3.28	1.34	.79 [.74; .84]	.86 [.83; .89]	.93 [.91; .95]	2.30	1.28	.89 [.83; .89]	.90 [.87; .92]	.90 [.87; .93]
6 M	3.41	2.31	.75 [.69; .80]	.86 [.83; .89]	.93 [.91; .95]	2.26	1.10	.78 [.73; .83]	.86 [.82; .89]	.85 [.79; .90]

*Note*: *W* women, *M* men

#### Internal consistency

Table 5 also presents Cronbach’s alpha coefficients of the Spanish ECR-12 and the original ECR (Brennan et al., 1998) subscales. Given that both of the Spanish ECR-12 subscales are comprised of a third (i.e., 6 out of 18) of the respective items in the original ECR, it is not surprising that the internal consistency Spanish ECR-12 subscales are systematically lower than those of the original ECR. Cronbach’s alpha coefficients varied from .72 to .83 for the brief anxiety subscale and from .78 to .89 for the brief avoidance subscale across samples, which suggested good internal consistency.

#### Evidence of concurrent validity

Table 1 displays the validity scales’ reliability coefficient and the standardized regression coefficients for all samples. As expected, both attachment-related anxiety and avoidance were associated with higher emotion regulation difficulties in community-based adult, student, and gay/lesbian adult samples. In samples 1 and 2, but not in sample 5, the Spanish ECR-12 subscale of anxiety appears to underestimate the association between this insecurity and emotion regulation difficulties, when its regression coefficients are compared with those of the original ECR anxiety subscale (*p*s < .01). As predicted, attachment-related avoidance was negatively associated with the two subscales of dyadic empathy in samples of adults from the community and students. In sample 1, but not sample 2, the Spanish ECR-12 subscale of avoidance appears to underestimate the association between this insecurity and perspective taking (i.e., cognitive empathy), when compared with the original ECR avoidance subscale (*p* < .01). Attachment-related anxiety was weakly related to lower perspective taking, but positively related to empathic concern (i.e., emotional empathy).

Both attachment-related anxiety and avoidance predicted higher levels of psychological distress (i.e., anxiety, depression, stress) and lower levels of life satisfaction across samples. In the community sample of couples, depression was more strongly related to attachment-related anxiety than avoidance for both men and women. No significant differences were found between the coefficients obtained with the original ECR and the Spanish ECR-12 for these measures, suggesting equivalence. Finally, relationship satisfaction was more strongly associated with attachment-related avoidance than anxiety in both samples 5 and 6.

## Discussion

The desire to form close emotional bonds with others is a universal human need that reflects the profoundness of the effect of attachment in well-being (Bowlby, 1982). To examine the nature of emotional bonds, and its correlates on mental health and well-being (Mikulincer & Shaver, 2016), it is necessary to have validated instruments that will assess this variable in different social and cultural contexts. The aim of the present study was to provide a significant contribution to the field by developing a new brief ECR (i.e., Spanish ECR-12) that would be appropriate to use with Chilean populations. This effort is considered relevant because validating the use of scores of one scale in a different context is always necessary, given that linguistic and cultural differences can be detected (Hambleton, Merenda, & Spielberg, 2005).

In that sense, the present work gives continuity to the study of adult romantic attachment in Latin American populations, with an instrument that overcomes the limitations of the previous short ECR versions. As demonstrated by the present study, the three existing ECR short versions were not suitable when applied to different Chilean samples because the factorial structure was not replicated. These results revealed that these versions would not be an appropriate representation of Chilean adults’ romantic attachment, supporting the aforementioned notion that the use of instruments in different contexts need to be empirically examined rather than assumed (Hambleton et al., 2005).

The appropriateness of this new measure was determined by examining the psychometric properties (i.e., factor structure, gender invariance, reliability, and concurrent validity) of the Spanish ECR-12 when administered to six samples of diverse Chilean populations. Our findings support the notion that the Spanish ECR-12 preserved the good psychometric properties of the 36-item Chilean ECR version (Spencer et al., 2013b) and the original ECR (Brennan et al., 1998).

Using statistical and theoretical criteria, six items from the original attachment anxiety subscale were selected (i.e., items 6, 8, 18, 20, 24, and 30) and six items from the original attachment avoidance subscale (i.e., items 5, 9, 13, 17, 23, and 27), preserving the two-dimensional structure of the original ECR (Brennan et al., 1998). These items are representative of all the theoretical components of attachment-related anxiety, but only two out of three components of attachment-related avoidance, as none of the retained Spanish ECR-12 item tapped the excessive need for self-reliance. It is possible that Chilean culture, characterized as a collectivistic society, is inconsistent with the reluctance to depend on others, a characteristic that is more frequently observed among individuals with greater attachment avoidance from more individualistic cultures (Friedman et al., 2010; Schmitt, 2010).

The Chilean culture may correspond to one of the types of collectivist cultures—the culture based on honor—characteristics of Mediterranean countries, belonging to Latin America, the Middle East, and Africa (Agishtein & Brumbaugh, 2013; Uskul, Oyserman, & Schwarz, 2010). These groups value having a good reputation and therefore a positive social image, which is maintained through social interdependence (Rodríguez-Mosquera, 2018).

The results of the CFA confirmed that the bidimensional structure of the Spanish ECR-12 was an adequate fit to the data in all five independent samples, with better indexes than the three existing brief versions. Our measure also proved to be invariant across gender. However, in the case of the lesbian and gay sample, two items from avoidance subscale were identified as not invariant (items 9 and 27). Both items refer to reluctance to self-disclose, being more representative of avoidance for gay men than lesbian. From a heteronormative point of view, gender shapes how men and women experience intimacy and self-disclosure of feelings toward the partner, with women expressing their personal feelings and their desire of intimacy more openly in their relationships (Elliott & Umberson, 2008; Peplau, 2001; Simon & Nath, 2004). Current knowledge about differences between gay men and lesbian in intimate relationships is still limited, but findings are in the same line: men in same-sex relationships are usually less likely to self-disclose and to communicate their feelings to their male partner than women are to their female partner (Peplau & Fingerhut, 2007; Umberson, Thomeer, & Lodge, 2015). And this aspect could be particularly salient among same-sex relationships, considering that communication has been identified as a central component of the relationship success among same-sex couples (Riggle, Rothblum, Rostosky, Clark, & Balsam, 2016). However, this is a tentative explanation that needs further exploration in future studies.

In addition, the Spanish ECR-12 proved to be a reliable measure in our six samples, with Cronbach’s alphas ranging from .72 to .83 for the attachment anxiety subscale and .78 to .89 for the attachment avoidance subscale. Although these coefficients were slightly lower than the alphas reported for the Chilean ECR version, this is an expected result given the reduction in the number of items.

Finally, attachment anxiety and attachment avoidance were consistently associated with several theoretically related variables (e.g., emotion regulation difficulties, dyadic empathy, psychological distress, life satisfaction, and relationship satisfaction), in a way that is in line with attachment theory and previous studies. Attachment anxiety encompasses a negative model of the self; this dimension is characterized by the fear of abandonment, dependence, and chronic doubts of one’s lovability, which is often associated with the hyperactivation of the attachment system, due to the dependency of others, and the excessive need for approval. Attachment avoidance encompasses a negative model of others; this dimension is characterized by discomfort with closeness, excessive self-reliance, and difficulty in trusting in others, which is often associated with the deactivation of the attachment system and expressed through their desire to physically and emotionally distance themselves from others (Bartholomew & Horowitz, 1991; Mikulincer & Shaver, 2016). It is well established that attachment insecurity (i.e., higher attachment anxiety and/or higher attachment avoidance) is associated with poor outcomes for the individual and couple’s functioning (Mikulincer & Shaver, 2016). More specifically, and in line with our results, attachment insecurity has been associated with higher emotion regulation difficulties (Guzmán-González et al., 2016, b; Han & Kahn, 2017), lower empathy (Lafontaine et al., 2016, b), more mental health problems and psychological distress (Mortazavizadeh & Forstmeier, 2018), lower life satisfaction (Tepeli Temiz & Comert, 2018), and lower relationship satisfaction (Li & Chan, 2012; Feeney, 2016).

However, it is worth noting, that for two of the attachment-related constructs that we tested, links were found in some of our samples only. First, perspective taking was not associated with attachment anxiety in the community sample (sample 1), but we found a small association in sample 2 composed of university students; the effect sizes were small in both samples. More perspective taking in university students was associated with lower anxiety, which is theoretically expected and in line with findings from previous studies (Lafontaine et al., 2016a). Second, relationship satisfaction was slightly associated with both attachment anxiety and attachment avoidance within gay and lesbian individuals (sample 5). Based on the sample of heterosexual couples (sample 6), attachment avoidance was also related to relationship satisfaction, but not attachment anxiety, for both men and women. This last finding differs from previous evidence showing that attachment anxiety is related to lower relationship satisfaction (Feeney, 2016), but this is line with what has been detected in Spanish and Chilean heterosexual couples (Heresi-Milad et al., 2013; Molero, Shaver, Fernández, Alonso-Arbiol, & Recio, 2016). This result can be explained due to the small sample size of the heterosexual couple sample or to the greater acceptance of attachment anxiety in this cultural context (Molero, Shaver, Ferrer, Cuadrado, & Alonso-Arbiol, 2010), demonstrating a less harmful effect on relationship satisfaction than attachment avoidance. In brief, attachment anxiety and attachment avoidance were generally related to attachment-related constructs in the expected way. However, in some samples, some associations were stronger (or not significant) than in other samples for these two constructs. These results demonstrate the importance of distinguishing between different samples and highlighting the effect of other relevant variables (e.g., age, status relationship) in future research.

The findings of this study can broaden the discussion of cultural aspects related to the evaluation of the attachment construct in diverse Spanish-speaking populations, and also contributes to the existing instruments available in this context (e.g., Valor-Segura, Expósito, & Moya, 2009). Although the relevance of the construct in different cultures is recognized, it is necessary to take into account that its expression may vary in different cultures and groups (e.g., LGBT), and that some of its dimensions may be underrepresented (Daouk-Öyry & Zeinoun, 2017). Theoretical and empirical development is needed to better understand the cultural and contextual aspects of attachment, as these aspects are scarcely addressed in a systematic way (Keller, 2013; Vicedo, 2017).

Limitations of the present study include the sole use of self-report measures; other measures such as behavioral observations could be included in future studies. In addition, the use of different methods of sample recruitment (e.g., snowball sampling, contacted by research assistants or email) and their potential effects on the results was not possible to explore in the present study. Recruitment strategies could be included as a confounding variable in future studies. This study included many validity criteria variables such as relationship satisfaction, emotional regulation, and psychological distress. However, to provide more evidence of concurrent validity, future studies could consider other relevant variables related to couple functioning (e.g., commitment and dependence toward the partner) that were not included in the present study.

## Conclusion

Despite its limitations, our findings supported the good psychometric properties of the Spanish ECR-12 across six diverse samples, such as university students, couples from the community, among others. In terms of its implications, the current study provides a suitable instrument that can be used by professionals or clinical working with individuals, couples, and families. In addition, this brief version can be a feasible tool for researchers conducting studies that require a large battery of test.

## Data Availability

The datasets generated and/or analyzed during the current study are not publicly available because it could compromise research participant consent, but are available from the corresponding author on reasonable request.

## Abbreviations

ECR: Experiences in Close Relationships
Spanish ECR-12: Spanish Experiences in Close Relationships 12 items
ECR-S: Experiences in Close Relationships Scale Short Form
ECR-12: Experiences in Close Relationships 12 items
Chilean B-ECR: Chilean brief version of the Experiences in Close Relationships
EFA: Exploratory factor analysis
RQ1: Research question 1
RQ2: Research question 2
RQ3: Research question 3
RQ4: Research question 4
RQ5: Research question 5
RQ6: Research question 6
RQ7: Research question 7
RQ8: Research question 8
DERS: Difficulties in Emotion Regulation Scale
IRIC: Interpersonal Reactivity Index for Couples
OQ-45: Outcome Questionnaire
DASS-21: Abbreviated Scales of Depression, Anxiety, and Stress
CES-D: Center for Epidemiologic Studies Depression Scale
RAS: Relationship Assessment Scale
KMO: Kaiser-Meyer-Olkin
CFA: Confirmatory factor analyses
CFI: Comparative fit index
TLI: Tucker-Lewis index
RMSEA: Root mean square error of approximation
